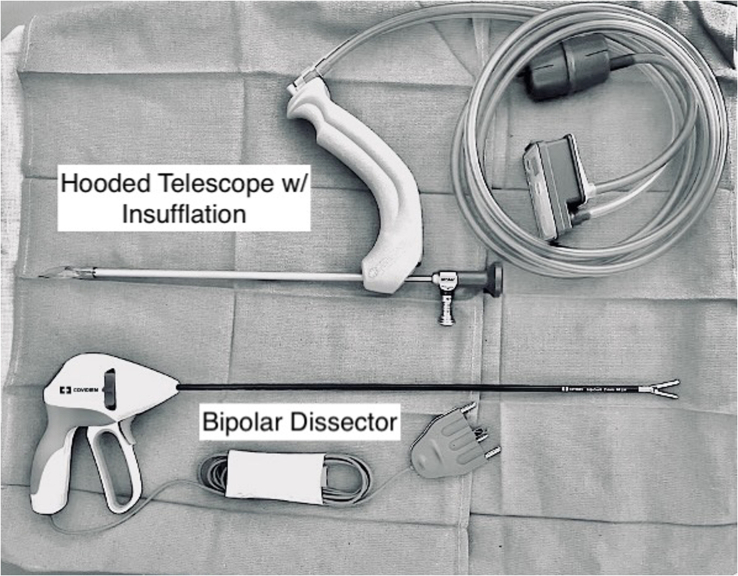# Transcervical Endoscopic Esophageal Mobilization: An Approach to Transhiatal Esophagectomy

**DOI:** 10.1016/j.atssr.2024.09.011

**Published:** 2024-09-28

**Authors:** Jennifer Livschitz, Joshua Melamed, Britton Donato, Amy Yoonjin Lee, Huaying Dong, Aniko Szabo, William B. Tisol, Paul L. Linsky, Mario G. Gasparri, David W. Johnstone

**Affiliations:** 1 Division of Cardiothoracic Surgery, Medical College of Wisconsin, Milwaukee, Wisconsin; 2 Department of Biostatistics, Beth Israel Deaconess Medical Center, Boston, Massachusetts; 3 Department of Biostatistics, Medical College of Wisconsin, Milwaukee, Wisconsin; 4 Department of Surgery, Advocate Aurora Health, Milwaukee, Wisconsin

## Abstract

**Background:**

Transcervical endoscopic esophageal mobilization (TEEM) is an approach to transhiatal esophagectomy that aims to minimize blood loss and decrease the operative time commonly associated with traditional transhiatal technique.

**Methods:**

A retrospective chart review was conducted on patients who underwent TEEM esophagectomy between 2009 and 2020. Baseline characteristics, perioperative outcomes, and postoperative complications were recorded. To report survival, a Kaplan-Meier survival plot was developed using SAS software (SAS Institute).

**Results:**

A total of 241 patients underwent TEEM esophagectomy. The mean operative time was 185.1 ± 59.3 minutes, blood loss was 251.4 ± 212.3 mL, the number lymph nodes dissected was 13.6 ± 6.2, and the length of stay was 11.9 ± 8.5 days. In the postoperative setting, 68 (28.2%) patients experienced hoarseness, 62 (25.7%) had postoperative atrial fibrillation, 30 (12.4%) had an anastomotic leak, and 12 (4.6%) experienced chylothorax. The overall 30- and 90-day mortality rates were 2.1% (5 of 241) and 4.6% (11 of 241), respectively. The median overall survival was 2.36 years, with 60% 3-year survival and 40% 5-year survival.

**Conclusions:**

TEEM esophagectomy is a safe approach with acceptable postoperative morbidity and mortality and shorter operative times compared with historical norms.

**In Short**

▪ TEEM esophagectomy is an efficient modification of transhiatal esophagectomy using a 2-team approach and modern endodissection tools.
▪ The technique reduces blood loss, operative times, and short-term mortality and provides long-term oncologic outcomes similar to those with other surgical approaches to esophageal resection. A consistent surgeon team appears to afford the best results.
▪ For experienced esophageal surgeons, the TEEM approach should require a short learning curve to obtain consistent results.

Esophageal cancer is an aggressive malignant disease with poor survival rates.^1^ For locally advanced esophageal cancer, surgical resection with esophagectomy remains a cornerstone of treatment.^2^ Esophagectomy can be performed by several different surgical techniques: transhiatal (TH), transthoracic (TT), and 3-hole (McKeown) techniques.^3^ The location of the tumor and the tumor’s size help determine which technique is most appropriate for a given patient, with specific strengths and weaknesses associated with each technique.^4^ Despite decades of surgical experience, an esophagectomy remains a time-consuming procedure with relatively high rates of morbidity and mortality.

Many studies have compared the various techniques, with no clear consensus on which technique produces the best oncologic outcome with the most acceptable morbidity profile.^5^, ^6^, ^7^ TH esophagectomy can be advantageous in avoiding TT dissection and has been shown to have oncologic outcomes equivalent to those of other approaches. However, TH esophagectomy traditionally involves a laparotomy and left cervical incision with “blind” dissection of the midesophagus which, although generally safe, can lead to high blood loss during this portion of the operation and increase the risk of complications such as tracheal and azygous injuries.

The thoracic surgical team at Froedtert Hospital and the Medical College of Wisconsin (Milwaukee, WI) developed an approach known as TH esophagectomy with transcervical endoscopic esophageal mobilization (TEEM). The TEEM approach uses a precise and time-sparing method for performing esophagectomies. We report a retrospective 10-year study with long-term follow-up conducted on all TEEM esophagectomies performed at the Medical College of Wisconsin and Froedtert Hospital.

## Material and Methods

A retrospective review was conducted on patients who underwent TEEM esophagectomy at Froedtert Hospital and the Medical College of Wisconsin between 2009 and 2020. Institutional Review Board approval for this project was obtained on October 21, 2020 (PRO00039300) with a waiver of informed consent given no direct contact with subjects. Baseline characteristics, including sex, age, body mass index, comorbidities (diabetes mellitus, hypertension, chronic obstructive pulmonary disease, hiatal hernia, and preoperative atrial fibrillation), tumor histologic type, and clinical and pathologic staging, were obtained. Additionally, perioperative, and postoperative outcomes, including operative time, blood loss, length of stay, total lymph nodes dissected, and postoperative complications (hoarseness, postoperative atrial fibrillation, vocal cord injection, vocal cord dysfunction at 6 months, anastomotic leak, chyle, 30-day mortality, and 90-day mortality), were also recorded. To report survival, a Kaplan-Meier survival plot was developed using SAS software (SAS Institute). Patients with benign disease were not included in the Kaplan-Meier survival curve. The American Joint Committee on Cancer seventh edition TNM manual was used for staging.

### Technique

Patients are admitted preoperatively for bowel preparation and a peripherally inserted central catheter line for laboratory testing and medications. Bowel preparation is performed routinely to prevent postoperative obstipation. After intubation, the patient is positioned supine with a nasogastric (NG) tube in place and the head extended and rotated to the right. The operation is performed through an upper midline laparotomy and an oblique low left cervical incision. Our practice is to have 2 surgeons working simultaneously, which significantly reduces the operative time. Once absence of peritoneal metastases is confirmed, the neck is opened, and the esophagus encircled with a Penrose drain for traction purposes. Esophageal dissection instruments are shown in the Supplemental Figure.

The abdominal operation is a standard gastric mobilization and lymphadenectomy, including the Kocher maneuver, division of the left gastric pedicle and short gastrics (LigaSure bipolar electrosurgical device, Medtronic), and resection of the proximal stomach, including the left gastric nodal tissue. A feeding jejunostomy is typically placed as well.

From the neck, after limited blunt mobilization of the cervical esophagus, circumferential mobilization of the esophagus is performed from the thoracic inlet to the diaphragm by using a hooded Sorin dissection scope with insufflation and a straight 5-mm LigaSure bipolar dissector (Video). This procedure is performed in 2 phases: the posterolateral planes with the esophagus retracted anteriorly in the neck, and the anterior and lateral planes with the esophagus retracted cephalad in the neck, by using the Penrose drain. Paraesophageal lymph nodes can be removed, and in some cases, subcarinal nodes can be seen and removed. The pleural envelopes are left largely intact, if possible, because they house the conduit in the midline after reconstruction.

**Figure 1:**
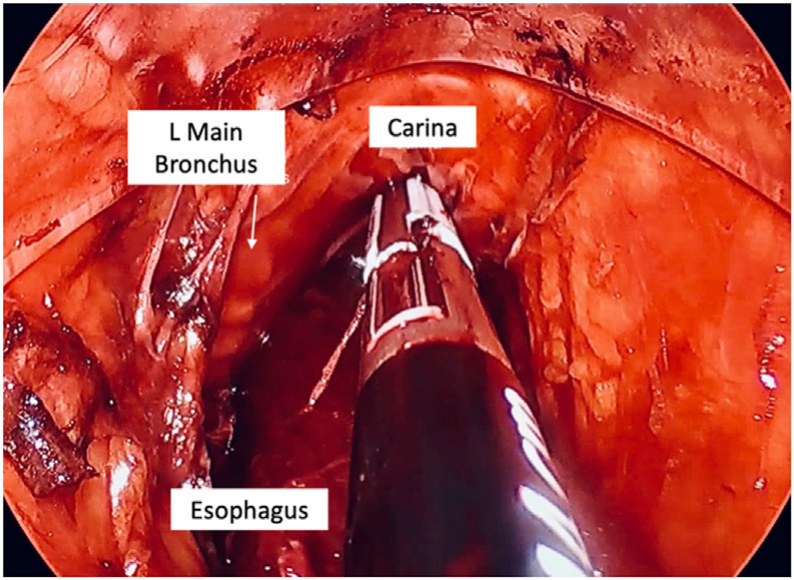
View of the anterior dissection plan at the level of the carina. The esophagus lies posterior to the dissector. (L, left.)

**Figure 2:**
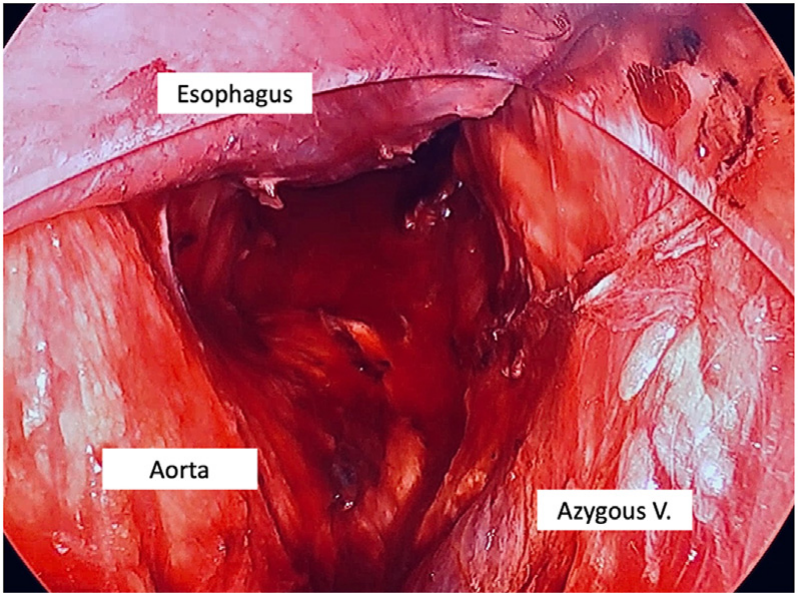
Completed posterior dissection at the level of the midesophagus, including a view of the descending aorta. The esophagus lies anterior to the scope hood. (V., vein.)

Once the 2 mobilizations are complete, the hiatus is opened from the abdomen, final dissection of the gastroesophageal junction and terminal esophagus is completed, and the esophagus is retracted into the neck for division with an endostapler. The proximal end is resected and submitted as final margin. The completed anterior and posterior dissection planes are shown in Figures 1 and 2.

A long Penrose drain is incorporated into this staple line to follow through the mediastinum as the esophagus is brought into the laparotomy space with the stomach; this is later used to pass a large Foley catheter from the neck to which the gastric conduit is fixed for translocation to the neck.

After the stomach is divided and the resection specimen is passed off the surgical field, the gastric conduit is housed in a condom-tipped ultrasound bag, sutured at its proximal end to the base of the condom flange and to the tip of a 32F Foley catheter passed from the neck incision into the hiatus. The condom tip is ligated above and below the inflated Foley balloon, and the conduit is then passed into the hiatus and brought into the cervical incision, where the housing bag is slipped off.

Assuming there is sufficient esophageal and gastric length, an overlay longitudinally oriented anastomosis is created with a 45-mm endostapler, and the resulting enterotomy between the esophagus and the stomach is closed transversely with a transverse anastomosing stapler after advancing the NG tube into the conduit. This final staple line is suture imbricated in Lembert fashion. The completed anastomosis is reduced into the prevertebral space, any mediastinal slack in the conduit is reduced from the abdomen, and the incisions are closed. We do not leave cervical drains. The NG tube is bridled at the nose, and the patient is extubated.

The typical postoperative course does not require intensive care unit admission at any point. The NG tube is removed, and enteral feedings begin on postoperative day 3 to 4 depending on bowel function. A contrast esophagram is performed on postoperative day 6, and barring complications, discharge is on postoperative day 7 to 8 on full liquids and nocturnal enteral supplements. A regular diet is started about 2 weeks after discharge, feedings are stopped, and the jejunostomy tube is removed about a month postoperatively.

## Results

A total of 241 patients underwent TEEM esophagectomy between 2009 and 2020. There were 230 patients with malignant disease, 195 (81%) with adenocarcinoma and 33 (14%) with squamous cell carcinoma, and 11 patients with benign disease, 8 (3%) with achalasia, 3 (1%) with stricture, and 2 (<1%) with high-grade dysplasia. In terms of comorbidities, a total of 144 (59.8%) patients received preoperative diagnoses of hypertension, 54 (22.4%) had a diagnosis of diabetes mellitus, 29 (11.6%) had a diagnosis of preoperative atrial fibrillation, and 25 (10.4%) had a diagnosis of chronic obstructive pulmonary disease. Intraoperative outcomes, including operative time, blood loss, and lymph node dissection, were reported. The mean operative time was 185.1 ± 59.3 minutes, mean blood loss was 251.4 ± 212.3 mL, and mean number lymph nodes dissected was 13.6 ± 6.2. The clinical characteristics, comorbidities, and oncologic characteristics of the patients included in the study are summarized in Supplemental Tables 1 and 2.

**Figure 3:**
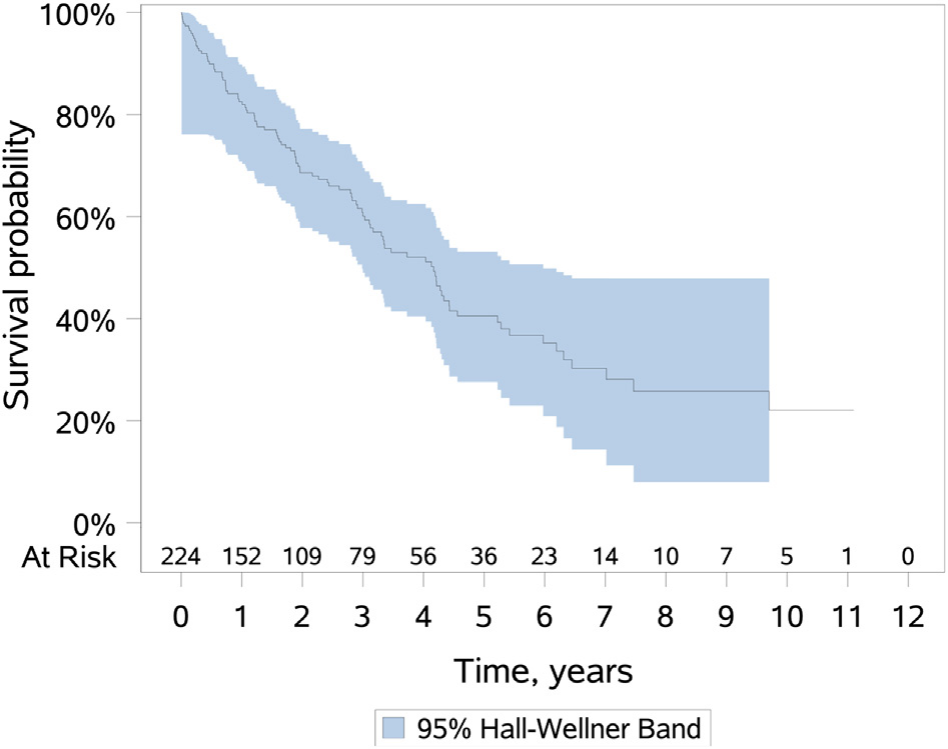
There was a median survival of 2.36 years, 3-year overall survival was 60.1%, and 5-year overall survival was 40.5%. The CI is 95%.

Postoperative outcomes included length of stay, esophageal anastomotic leak, hoarseness, vocal cord dysfunction, postoperative atrial fibrillation, chylothorax, 30-day mortality, and 90-day mortality (Table). The mean length of stay was 11.9 ± 8.5 days. Anastomotic leak occurred in 12.4% (30 of 241) of cases. Of the 30 anastomotic leaks, 9 patients required debridement or repair, 11 patients underwent stent placement, and 10 were managed nonoperatively. A total of 28.2% (68 of 241) of patients experienced hoarseness and 21.2% (51 of 241) had their vocal cords injected postoperatively, with 6.7% (16 of 241) experiencing long-term vocal cord dysfunction at 6 months. Postoperative atrial fibrillation occurred 25.7% (62 of 241), with a significantly higher rate in the early cohort compared with the later cohort (*P* = .0144). Perioperative amiodarone was administered routinely on the basis of institutional experience. Chylothorax occurred in 4.6% of patients and was managed with thoracic duct embolization when conservative measures were unsuccessful. The overall 30- and 90-day mortality rates were 2.1% (5 of 241) and 4.6% (11 of 241), respectively. The median overall survival was 2.36 years, with 60% 3-year survival and 40% 5-year survival (Figure 3).

**Table:** Perioperative and Postoperative Outcomes After Transcervical Endoscopic Esophageal Mobilization Esophagectomy

Variables	Total N = 241 (col %)
Operative time, min	185.1 ± 59.3
Blood loss, mL	251.4 ± 212.3
Length of stay, d	11.9 ± 8.5
Total lymph nodes, n	13.6 ± 6.2
Postoperative complications^a^	
Hoarseness	68 (28.2)
Postoperative atrial fibrillation	62 (25.7)
Vocal cord injection	51 (21.2)
Vocal cord dysfunction at 6 mo	16 (6.64)
Leak	30 (12.4)
Chyle	12 (4.56)
30-d mortality	5 (2.07)
90-d mortality	11 (4.56)

Values are mean ± SD.

a Patients can have more than 1 complication.

## Comment

Esophagectomy remains a relatively major and morbid procedure of those commonly performed by thoracic surgeons. The TEEM approach was developed as a way to reduce the time and morbidity of TH esophagectomy at a time when minimally invasive esophagectomy was still gaining traction in the thoracic surgical community. The advantages of this approach, such as the use of simultaneous cervicothoracic and abdominal surgeons, include a reduction in operative time, minimal blood loss, with short- and long-term results comparable to those of other approaches. However, some concerns remain, most notably the incidence of vocal cord weakness with this approach.

In this study, there was a mean operative time of 185 minutes, significantly shorter than historical institutional norms with TH esophagectomy, in large part the result of having a 2-team simultaneous approach. Additionally, there was a mean operative blood loss of 251 mL. In their extensive review of the TH technique, Orringer and colleagues^8^ reported an average intraoperative blood loss of 368 mL. With use of the endoscopic vein harvester in combination with insufflation and a bipolar cautery device, bleeding is minimized. Finally, this study reported 3- and 5-year survival of 60.1% and 40.5%, respectively, in patients undergoing surgery for malignant disease. This is comparable to the rates published in the literature. For example, in a large population-based study from Finland, Junttila and colleagues^9^ observed a 5-year survival of 39.3% after TH esophagectomy and 45.0% after TT esophagectomy.

The incidence of vocal cord palsy is an ongoing concern with the TEEM approach. Postoperative hoarseness was present in 28% of the patients included in this study. All patients with hoarseness beyond postoperative day 2 were evaluated by the otolaryngology department to assess for vocal cord paralysis and underwent vocal cord injection to minimize aspiration risk. Only 6.6% of the patients included in this study had persistent dysfunction at 6 months postoperatively, defined as the patient having persistent hoarseness, with repeat laryngoscopy showing persistent vocal cord paralysis. The cause of vocal cord palsy may be related to traction required on the esophagus, energy delivered in the periesophageal planes, or exposure techniques in the neck. The rates of vocal cord palsy declined in the last 3 years of this experience with the same pair of surgeons performing all cases. Additionally, during the first 4 to 5 years of this series, an ultrasonic dissector was used; this was changed to a bipolar device that was thought to minimize heat transfer around the esophagus. We do not recommend this approach for large tumors near the distal trachea and carina, or in cases where there is significant scarring anticipated in the mediastinum. In these cases, we typically perform either an Ivor Lewis or McKeown (3-hole) esophagectomy.

There is a modest learning curve associated with using the cervical dissection instruments, largely related to exposure. We have found that our fellows can become proficient in this technique after 5 to 10 cases, but this may vary by surgeon and surgeon experience. Surgeons who already have mature experience with esophagectomies by other approaches will find the learning curve relatively short because the knowledge of mediastinal relations and tissue tolerances is already established. Our strong impression from this experience is that the TEEM approach has benefits for our patients undergoing esophageal resection, in large part because of the reduction of operative times to 3 hours or less, a standardized operative plan, decreased blood loss, lower fluid requirements, and elimination of intensive care unit stays. Our length of stay is dictated by resumption of oral intake, barring complications, not by pain or mobilization issues related to the laparotomy. The technique is oncologically sound, with survival results comparable to those with other approaches.

## Figures and Tables

**Supplementary Figure 1:**