# Supplementary material for: Transcervical Endoscopic Esophageal Mobilization: An Approach to Transhiatal Esophagectomy
Source: Ann Thorac Surg Short Rep. 2024 Sep 28;3(1):201–5. doi: 10.1016/j.atssr.2024.09.011 (PMC11910823; doi:10.1016/j.atssr.2024.09.011)
Supplement: Supplementary Table 1 [file mmc2.docx]

**Supplemental Table 1:** Characteristics of Study Population

| **Variables** | **Total N=241 (col %)** |
| --- | --- |
| **Sex** |  |
| Female | 39 (16.2) |
| Male | 202 (83.8) |
| **Age** |  |
| Mean ± SD (years) | 62.7± 9.49 |
| **BMI** |  |
| Mean ± SD | 28.0 ± 6.8 |
| **Histology** |  |
| **Malignant** |  |
| Adenocarcinoma | 195 (80.9) |
| Squamous Cell Carcinoma | 33 (13.7) |
| High Grade Dysplasia | 2 (0.830) |
| **Benign** |  |
| Achalasia | 8 (3.32) |
| Stricture | 3 (1.24) |
| **Comorbidities*** |  |
| Diabetes Mellitus | 54 (22.4) |
| Hypertension | 144 (59.8) |
| COPD | 25 (10.4) |
| Hiatal Hernia | 13 (5.4) |
| Preoperative Atrial Fibrillation | 28 (11.6) |
| *Subjects can have more than one comorbidites | |
