# Supplementary material for: Transcervical Endoscopic Esophageal Mobilization: An Approach to Transhiatal Esophagectomy
Source: Ann Thorac Surg Short Rep. 2024 Sep 28;3(1):201–5. doi: 10.1016/j.atssr.2024.09.011 (PMC11910823; doi:10.1016/j.atssr.2024.09.011)
Supplement: Supplementary Table 2 [file mmc3.docx]

**Supplemental Table 2:** Oncologic Characteristics of Study Population

| **Variables** | **Total**  **N (col%)** |
| --- | --- |
| **Clinical TNM Stage** |  |
| **Adenocarcinoma** | **N=195** |
| Stage 0 | 1 (0.513) |
| Stage I | 24 (12.3) |
| Stage II | 18 (9.23) |
| Stage III | 125 (64.1) |
| Stage IV | 27 (13.8) |
| **Squamous Cell Carcinoma** | **N=33** |
| Stage 0 | 1 (3.03) |
| Stage I | 3 (9.09) |
| Stage II | 13 (39.4) |
| Stage III | 13 (39.4) |
| Stage IV | 3 (9.09) |
| **Pathological TNM Stage** |  |
| **Adenocarcinoma** | **N=195** |
| Stage 0 | 47 (24.1) |
| Stage I | 78 (40.0) |
| Stage II | 23 (11.8) |
| Stage III | 39 (20.0) |
| Stage IV | 8 (4.1) |
| **Squamous Cell Carcinoma** | **N=33** |
| Stage 0 | 9 (27.3) |
| Stage I | 10 (30.3) |
| Stage II | 6 (18.1) |
| Stage III | 6 (18.1) |
| Stage IV | 2 (6.06) |
