# Supplementary material for: Transcervical Endoscopic Esophageal Mobilization: An Approach to Transhiatal Esophagectomy
Source: Ann Thorac Surg Short Rep. 2024 Sep 28;3(1):201–5. doi: 10.1016/j.atssr.2024.09.011 (PMC11910823; doi:10.1016/j.atssr.2024.09.011)
Supplement: Supplementary Figure [file mmc4.docx]

Supplemental Figure Legend

Supplemental Figure. Instruments for transcervical esophageal mobilization including hooded 5 mm telescope with insufflation and long 5 mm bipolar dissector.
